# Involvement of calmodulin in regulation of primary root elongation by *N*-3-oxo-hexanoyl homoserine lactone in *Arabidopsis thaliana*

**DOI:** 10.3389/fpls.2014.00807

**Published:** 2015-01-13

**Authors:** Qian Zhao, Chao Zhang, Zhenhua Jia, Yali Huang, Haili Li, Shuishan Song

**Affiliations:** ^1^Department of Bioengineering, Biology Institute, Hebei Academy of SciencesShijiazhuang, China; ^2^Hebei Engineering and Technology Center of Microbiological Control on Main Crop DiseaseShijiazhuang, China

**Keywords:** quorum sensing, calmodulin, *N*-acyl-homoserine lactone, signal transduction, plant development

## Abstract

Many bacteria use signal molecules of low molecular weight to monitor their local population density and to coordinate their collective behavior in a process called “quorum sensing” (QS). *N*-acyl-homoserine lactones (AHLs) are the primary QS signals among Gram-negative bacteria. AHL-mediated QS plays an essential role in diverse bacterial physiological processes. Recent evidence shows that plants are able to sense bacterial AHLs and respond to them appropriately. However, little is known about the mechanism by which plants perceive and transduce the bacterial AHLs within cells. In this study, we found that the stimulatory effect of *N*-3-oxo-hexanoyl homoserine lactone (3OC6-HSL) on primary root elongation of Arabidopsis was abolished by the calmodulin (CaM) antagonists *N*-(6-aminohexyl)-5-chloro-1-naphthalene sulfonamide (W-7) and trifluoperazine (TFP). Western-blot and ELISA analysis revealed that the concentration of CaM protein in Arabidopsis roots increased after treatment with 1 μM 3OC6-HSL. Results from quantitative RT-PCR demonstrated that the transcription of all nine *CaM* genes in Arabidopsis genome was up-regulated in the plants treated with 3OC6-HSL. The loss-of-function mutants of each *AtCaM* gene (*AtCaM1-9*) were insensitive to 3OC6-HSL-stimulation of primary root elongation. On the other hand, the genetic evidence showed that CaM may not participates the inhibition of primary root length caused by application of long-chained AHLs such as C10-HSL and C12-HSL. Nevertheless, our results suggest that CaM is involved in the bacterial 3OC6-HSL signaling in plant cells. These data offer new insight into the mechanism of plant response to bacterial QS signals.

## Introduction

Bacteria sense their population density and act in concert using signal molecules of low molecular weight in a process termed “quorum-sensing” (QS) (Bassler, 1999; Fuqua et al., 2001; Taga and Bassler, 2003). These signal molecules, also called autoinducers, are often *N*-acyl homoserine lactones (AHLs) in gram-negative bacteria (Fuqua and Greenberg, 2002). Bacterial QS plays a central role in a variety of bacterial behaviors including biofilm production, induction of bioluminescence, antibiotic production and virulence factor expression (Miller and Bassler, 2001; Quiñones et al., 2005). In recent years, evidence has accumulated that plants are able to detect bacterial QS signal molecules and respond to these signals with altered gene expression or modification in development. The first evidence for the promoting effects of AHLs on plant root growth is reported by von Rad et al. (2008). They found that inoculation of Arabidopsis roots with *N*-hexanoyl homoserine lactone (C6-HSL) led to increased root elongation (von Rad et al., 2008). Thereafter, a growing body of evidence showed that AHLs can regulate primary root growth in a dose- and structure-dependent manner. The short-chained AHLs such as *N*-butyryl homoserine lactone (C4-HSL), *N*-hexanoyl homoserine lactone (C6-HSL), *N*-3-oxo-hexanoyl homoserine lactone (3OC6-HSL) and *N*-3-oxo-octanoyl homoserine lactone (3OC8-HSL) within concentration of 1–10 μM promote root growth of Arabidopsis (von Rad et al., 2008; Jin et al., 2012; Liu et al., 2012). However, AHLs with longer side-chains influence root growth in a manner different from short-chain AHLs. *N*-3-oxo-decanoyl homoserine lactone (3OC10-HSL) can induce the formation of adventitious roots in mung beans (Bai et al., 2012). C10-HSL and 3OC12-HSL are found to have strong activity in inhibiting root growth and stimulating lateral root and root hair development when more than 50 μM AHLs are applicated (Ortiz-Castro et al., 2008, 2011). Furthermore, accumulating evidence indicated that water-soluble short-chain AHLs are actively taken up into plant roots and transported along the roots into the shoot; in contrast, the lipophilic long-chain AHLs can't be transported in barley and Arabidopsis (Götz et al., 2007; von Rad et al., 2008; Sieper et al., 2014). More recently, Palmer et al. reported that the root growth effects of AHLs with long aliphatic acyl groups are dependent upon AHL amidolysis by a plant-derived fatty acid amide hydrolase (FAAH) to yield L-homoserine (Palmer et al., 2014). On the other hand, some AHLs or AHL-producing bacteria induced resistance against microbial pathogens (Schuhegger et al., 2006; Schikora et al., 2011; Schenk et al., 2012, 2014; Zarkani et al., 2013). 3OC14-HSL has been shown to induce resistance against biotrophic and hemibiotrophic pathogens in Arabidopsis and barley (Schikora et al., 2011). Similarly, resistance against *Pseudomonas syringae* induced by *Sinorhizobium meliloti* in Arabidopsis plants depended on the accumulation of AHL (Zarkani et al., 2013). Transcriptomic and proteomic analysis showed that treatments of plant with bacterial AHLs elicit significant changes in gene transcription and protein expression (Mathesius et al., 2003; Ortiz-Castro et al., 2008; Miao et al., 2012; Schenk et al., 2014). Arabidopsis mutants like *drr1* and *dhm1* that are tolerant or hypersensitive to alkamides from plants show alterations in primary and lateral root development and in root responses to C10-HSL, indicating that medium-chained AHLs and alkamides act through common signaling mechanisms (Morquecho-Contreras et al., 2010; Pelagio-Flores et al., 2013). All these data indicated that AHLs might mediate the interaction between bacteria and their host plant (Hartmann et al., 2014). However, the signal transduction pathways of AHLs in plant cells are widely unexplored so far. The identification and investigation of those components in signaling will be crucial to understand the mechanism of plant response to bacterial AHLs.

Calcium is an ubiquitous second messenger in plants. Intracellular cytosolic free Ca^2+^ concentration ([Ca^2+^]_i_) often shows significant elevation in response of plant to various environmental stimuli (Knight et al., 1991; van der Luit et al., 1999). Ca^2+^ signals are sensed and translated into proper cellular response by diverse Ca^2+^ binding proteins and their downstream targets. Calmodulin (CaM) is an acidic Ca^2+^ binding protein that possesses EF hand motifs, a helix-loop-helix structure for binding one Ca^2+^ ion. Upon Ca^2+^ binding, CaM undergoes conformational changes that promote either its own catalytic activity or its interaction with target proteins (Chin and Means, 2000; Snedden and Fromm, 2001; Cheval et al., 2013). Ca^2+^-CaM signaling has been implicated in various developmental and adaptation responses. Many exogenous and endogenous factors including light, temperature, drought and salt stress, pathogen-derived molecules and phytohormones provoke elevation in [Ca^2+^]_i_ of plants (Sander et al., 1999). Likely, the level of CaM was up-regulated by heat-shock (HS) in maize seedlings (Gong et al., 1997). Pharmocological analysis revealed that CaM participated in signal transduction in lemon in response to bacterial pathogen *Alternaria alternata* (Ortega et al., 2002). Studies of plants demonstrated the presence of multiple *CaM* gens that encode numerous CaM isoforms (Lee et al., 1995; Takezawa et al., 1995; Yang et al., 1996; Snedden and Fromm, 2001; Yamakawa et al., 2001; Zielinski, 2002). Braam and Davis (1990) reported that the expression of many plant *CaM* genes are induced by rain, wind and touch. Among of eight *CaM* genes in potato plants, *PCM1* showed an increase in expression upon touching and *PCM6* during tuberization (Takezawa et al., 1995). Soybean *CaM* genes *SCaM-4* and *SCaM-5* are reported to be required for defense against pathogen attack (Heo et al., 1999). *AtCaM3*, one of nine Arabidopsis *CaM* genes, has been shown to be involved in Ca^2+^-CaM mediated HS signal transduction pathway (Liu et al., 2003, 2005; Zhang et al., 2009). Recently, we have reported evidence for a transient elevation in [Ca^2+^]_i_ upon the exposure of Arabidopsis roots to C4-HSL (Song et al., 2011). Given that CaM, as a Ca^2+^ sensing protein, translates Ca^2+^ to downstream protein targets in numerous signal transduction cascades, CaM might be involved in bacterial AHL signaling in plant cells. However, there is no direct evidence for the participation of CaM in AHL-mediated primary root elongation in plants.

In this paper, we first demonstrated the involvement of CaM in 3OC6-HSL mediated primary root growth in Arabidopsis using pharmacological approaches. Then we observed the increased level of CaM protein and CaM gene transcription after treatment with 3OC6-HSL. Furthermore, we used T-DNA knockout mutants to provide genetic evidence for the role of individual CaM gene in primary root elongation regulated by 3OC6-HSL. A possible regulatory model of Ca^2+^-CaM in AHL signaling in plant cells is discussed.

## Materials and methods

### Plant materials and growth conditions

*Arabidopsis thaliana (L.)* cv. Columbia-0, the T-DNA insertion null mutants *cam1* (CS872565), *cam5* (SALK_007371C), *cam6* (SALK_071609C), *cam7* (SALK_074336C), *cam8* (SALK_022524C) and *cam9* (SALK_040392) purchased from ABRC (http://abrc.osu.edu), and *cam2, cam3*, and *cam4* generously gifted from Prof. Sujuan Cui in Hebei Normal University were used in this study. All seeds were surface-sterilized by 75% (v/v) ethanol for 30 s and 20% (v/v) NaClO for 5 min. After five washes with sterile distilled water, seeds were geminated on agar plates containing Murashige and Skoog (MS) medium (Murashige and Skoog, 1962) adjusted to pH 5.8. The plates were stratified at 4°C for 2 days and then placed in a plant growth chamber with a photoperiod of 12 h of light, 12 h of darkness, light intensity of 100 μmol·m^2^·s^−1^ and temperature of 22 ± 2°C. For primary root growth assay, the seeds were grown vertically for 3 days on MS agar plates, and the seedlings with similar root length were transferred to 1/2 MS agar plates containing the indicated compounds. For qRT-PCR assay, a hydroponic system was employed to cultivate Arabidopsis. Seedlings germinated on MS agar plates for 10 days were transplanted into a sterile plastic basin (18 by 11 cm) containing 500 ml of sterile Hoagland medium. After 7 days, the seedlings were used for compounds treatments. Plants grown under these conditions were more vigorous than those grown in potting media, uniformly absorbing solution with AHL. To ensure that the hydroponic system is free of bacterial contamination, the medium and compound solution were sterilized by passing them through a 0.22-μm filter just prior to use. The untreated plants were taken as the control. After harvest, an aliquot of the growth medium was plated on bacterial growth medium and incubated overnight to check for contaminations that occurred during the experiment. Any contaminated samples were discarded. For mutants identification assay, seedlings germinated on MS agar plates for 10 days were transplanted into vermiculite media and watered Hoagland medium once a week. After 1 month, the leaves of seedlings were harvested for RT-PCR.

### Compounds

*N*-(β-ketocaproyl)-DL-homoserine lactone (3OC6-HSL), *N*-decanoyl-DL-homoserine lactone (C10-HSL) and *N*-dodecanoyl-DL-homoserine lactone (C12-HSL) were purchased from Sigma-Aldrich (Taufkirchen, Germany), stored dry and diluted as 10 mM stock solutions in dH_2_O or ethanol just prior to use. Trifluoperazine (TFP) was purchased from Sigma (USA) and diluted as 10 mM stock solutions in methanol. *N*-(6-aminohexyl)-5-chloro-1-naphthalene-sulfonamide (W-7) was purchased from Santa Cruz Biotechnology (USA) and diluted as 10 mM stock solutions in dH_2_O. *N*-(6-aminohexyl)-1-naphthalene-sulfonamide hydrochloride (W-5) was purchased from Tokyo Chemical Industry (TCI, Japan) and diluted as 10 mM stock solutions in dH_2_O. All compound solutions were sterilized by passing them through a 0.22-μm filter.

### Pharmacological assay

CaM antagonists TFP, W-7 and W-5 were used for pharmacological assay. W-5 is the dechlorinatd analog of W-7 and is often used as control compound for understanding the specificity of W-7. After germination for 3 days, Arabidopsis (Columbia, Col-0) seedlings with similar root length were transferred to 1/2 MS medium plates containing the indicated concentrations of 3OC6-HSL or/and CaM antagonists. The untreated plants were taken as the control. Seedlings were grown vertically with the same growth condition as described above, and primary root length was assessed with a ruler after 7 days. The concentration of TFP used in this study ranged from 1 to 50 μM, while the concentration of W-7 and W-5 ranged from 30 to 200 μM. 1 μM 3OC6-HSL was used in all experiments. The results were normalized to the root length of control samples. Four to six independent experiments were done and data shown are the average of 30 samples.

### Immunodetection of CaM

Seventeen-day-old Arabidopsis Col-0 (Columbia, Col-0) seedlings by hydroponic culture were treated with 1 μM 3OC6-HSL. The untreated plants were taken as the control. Roots were harvested at 0, 1, 2, 3, 6, 12, and 24 h after 3OC6-HSL treatment and powdered with liquid nitrogen. Total protein were extracted with protein extracting buffer [50 mM Tris-HCl (pH 7.5), 5 mM EDTA, 5 mM EGTA, 10 mM DTT, 10 mM Na3VO4, 10 mM NaF, 1 mM PMSF, 2 μg/ml Pepstain, 2 μg/ml Leupeptin, 2 μg/ml Aprotintin, 10% (v/v) NP-40, 10 mM β-Glycerophosphate] after 14,000 rpm centrifuge for 20 min at 4°C. Protein was quantified by the Bradford method using bovine serum albumin as the standard (Bradford, 1976). For ELISA analysis, 2 μg total proteins were coated in the 96-well plate, and the polyclone antidoby against wheat CaM (generous gift from Prof. Daye Sun in Hebei Normal University) and HRP-labeled goat anti-rabbit IgG antibody (Bioworld Tchnology Inc., St. Louis Park, USA) were used to determine the level of CaM. The results were normalized to the CaM protein level of untreated samples. For Western blot analysis, total proteins were separated on 12% SDS-polyacrylamide gel, transferred to PVDF film, hybridized with polyclonal antibody against wheat CaM, and detected the accumulation of CaM by HRP-labeled goat anti-rabbit IgG antibody (Bioworld Tchnology Inc., St. Louis Park, USA). The images were detected by Biodlight ECL Chemiluminescent HRP Kit (Bioworld Technology Co. Ltd, St. Louis Park, USA) and scanned by BIO-RAD ChemiDoc TM MP Imaging System (Bio-Rad Laboratories, Inc. USA). Actin was used as the internal quantification control by using plant actin mAb (Bioworld Tchnology Inc., St. Louis Park, USA).

### Quantitative real-time PCR (qRT-PCR)

Seventeen-day-old seedlings were cultured in Hoagland medium with or without 1 μM 3OC6-HSL. Roots of the treated plants were harvested at 0, 1, 2, 3, 6, 12, and 24 h after 3OC6-HSL treatment and total RNA was extracted from the treated and untreated roots using the RNAiso Plus reagent (TaKaRa, Dalian, China) according to the manufacturer's instructions. The cDNA was synthesized using the PrimeScript® RT Reagent Kit with gDNA Eraser (TaKaRa, Dalian, China) according to the manufacturer's instructions. For the relative quantification of gene expression, the comparative C_*T*_ method (Livak and Schmittgen, 2001) was used with the 7500 Real Time PCR System (Applied Biosystems, Foster City, CA, USA). For qRT-PCR, received cDNA was diluted 15 times in diethylpyrocarbonate-treated water. PCR amplification was done in a total volume of 20 μl containing 5 μl of diluted cDNA, 0.4 μl of each primer (10 μM), and 10 μl of SYBR Premix Ex Taq (TaKaRa, Shiga, Japan). The following qRT-PCR thermal cycling program was employed: 10 s at 95°C and 40 cycles for 5 s at 95°C and 31 s at 60°C. The amount of target was normalized to the endogenous reference gene *Actin 2*. Values were normalized to the 0 h time point. For technical control, each qRT-PCR experiment was repeated four times on the same 96-well plate. Each data point represents the average of three independent experiments. A 1.5-fold increase (ratio > 1.5) or 1.5-fold decrease (ratio < 0.8) in expression in the treated plants compared with the untreated plants was considered as up-regulation or down-regulation related to 3OC6-HSL response. The specific primers for qRT-PCR are shown in Supplementary Table 1.

### Identification of CaM null mutants

The leaves of one-month-old wild type (Columbia, Col-0) and CaM mutants (*cam1-cam9*) seedlings cultured in vermiculite were harvested respectively and total RNA was extracted using the RNAiso Plus reagent (TaKaRa, Dalian, China) according to the manufacturer's instructions. The cDNA was synthesized using the PrimeScript® RT Reagent Kit with gDNA Eraser (TaKaRa, Dalian, China) according to the manufacturer's instructions. For RT-PCR, amplification was done in a total volume of 20 μl containing 1 μl of received cDNA, 1 μl of each primer (10 μM), 2 μl of 10×PCR buffer, 2 μl of dNTP (2.5 mM each), 0.2 μl of rTaq DNA Polymerase (5 units/μl, TaKaRa, Shiga, Japan) and 12.8 μl of sterilized distilled water. The following RT-PCR thermal cycling program was employed: initial denaturation for 5 min at 94°C, 30 cycles for 40 s at 94°C, 50 s at 52°C and 1 min at 72°C, and then extension for 10 min at 72°C. Actin 2 was used as the endogenous reference gene. The primer pairs were designed at 5′-UTR and 3′-UTR respectively for containing ORF sequence of each CaM genes and avoiding homologos mismatches between the nine CaM genes (Supplementary Table 2).

### Analysis of primary root growth

The seeds of wild type (Columbia, Col-0) and mutants *Atcam1-9* (*cam1-cam9*) germinated at MS agar plate vertically. After 3 days, seedlings with similar root length were transferred to 1/2 MS agar plates containing 1 μM 3OC6-HSL or 50 μM C10-HSL or 50 μM C12-HSL. Because of the space limitation in one plate, the seedlings of WT and each three mutants were grown side by side vertically in one plate with the same growth condition as described above. The equivalent dH_2_O-treated plants and ethanol-treated plants were taken as the solvent control. Primary root length was assessed with a ruler. In addition, the fresh weight of seedlings were assessed with an electronic balance (accuracy of 10,000 g) after 7 days. Each experiment included at least 30 seedlings for each genotype and treatment. Data shown are the average of four independent experiments.

For dissecting the root elongation, the length of root meristem zone and root elongation zone were measured on the 7-days-old seedlings fixed in ethanol: acetic acid (3:1) and mounted in choral hydrate. Mocroscopy was performed on a Leica DM4000 B (CMS GmbH, Wetzlar, Germany) equipped with a Leica DFC 420C camera. Root meristem zone length was assessed as the distance between the quiescent center and the first elongating cell with a vacuole. The length of elongation zone was measured as the distance from the first elongating cell to the first cell with a root hair. The number of the cortical cells in root meristem and elongation zone was obtained by counting the cortical cells under the high-resolution images of the root tips using Image J software (http://rsb.info.nih.gov/ij/). The length of cortical cells in the root meristem or elongation zone was calculated by dividing the root meristem or elongation zone length by the number of the cortical cells in root meristem or elongation zone. At least 20 seedling were processed, and at least three independent experiments were conducted, giving the similar statistically significant results.

### Statistical analysis

For all experiments, the overall data were statistically analyzed in the DPS v7.05 program. Univariate and multivariate analyses with a Duncan's new multiple range method were used for testing different root growth responses of different ecotype, wild-type, and CaM mutant lines to 3OC6-HSL or to CaM antagonists.

## Results

### CaM antagonists block the impact of 3OC6-HSL on primary root length

**Figure 1:**
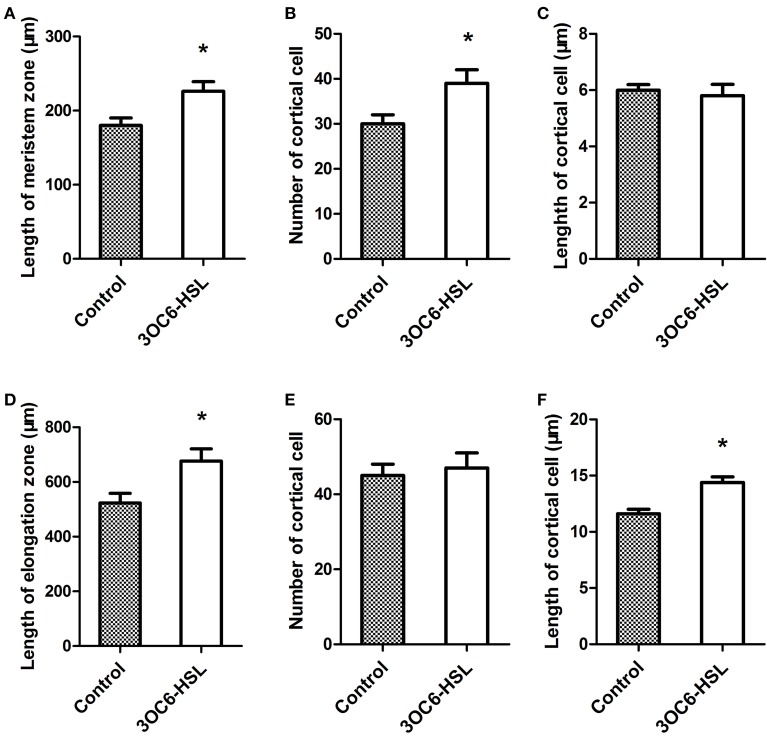
**Effects of 3OC6-HSL on Arabidopsis root meristem zone and root elongation zone**. The length of the root meristem zone **(A)**, the number of cortical cells in the meristem zone **(B)**, the length of the root elongation zone **(D)** and the number of the cortical cells in elongation zone **(E)** were measured on the wild type seedlings grown on vertically oriented plates containing with or without 1 μM 3OC6-HSL after 7 days of cultivation. The length of cortical cell in the root meristem zone **(C)** and in the root elongation zone **(F)** were calculated by dividing the root meristem or elongation zone length by the number of the cortical cells in the root meristem or elongation zone. Data shown are the average of three independent experiments giving the similar statistically siginificant results. Each experiment included at least 20 seedlings. Entries with *p*-values < 0.05 shown with asterisk.

A previous study showed that 3OC6-HSL promotes the primary root elongation of wild-type Arabidopsis (Jin et al., 2012; Liu et al., 2012). Root growth is controlled by the cell division in meristem zone and the cell expansion in elongation zone. In order to dissect the promoting effect of 3OC6-HSL on primary root elongation, we measured the length of meristem and elongation zones in primary roots 7 days after application of 3OC6-HSL to Arabidopsis root systems. The results revealed that the primary roots of plants treated with 3OC6-HSL had both longer meristem and elongation zones than those of solvent-treated wild type plants (Figures 1A,D). The difference in the length of elongation zone (30.2%) was much more pronounced than the difference in the length of meristem zone (13.6%). To further pinpoint the process whereby 3OC6-HSL functions, we counted the number and calculated the length of cortical cells in the meristem and elongation zone. We found that treatment with 3OC6-HSL caused a 30% increase in the number of cortical cell in the meristem zone compared to the solvent-treated roots while no significant increase in the length of cortical cells in the meristem zone was observed after 3OC6-HSL treatment, indicating that the increase in meristem size is mainly due to the increased number of cortical cells in root meristem (Figures 1B,C). On the other side, analysis for root elongation zone demonstrated that the elongated root elongation zone induced by 3OC6-HSL contained similar number of cortical cells but longer cells than the control, suggesting an increase in cell expansion (Figures 1E,F). Collectively, these results suggest that 3OC6-HSL promotes primary root growth via changes in cell division and elongation.

**Figure 2:**
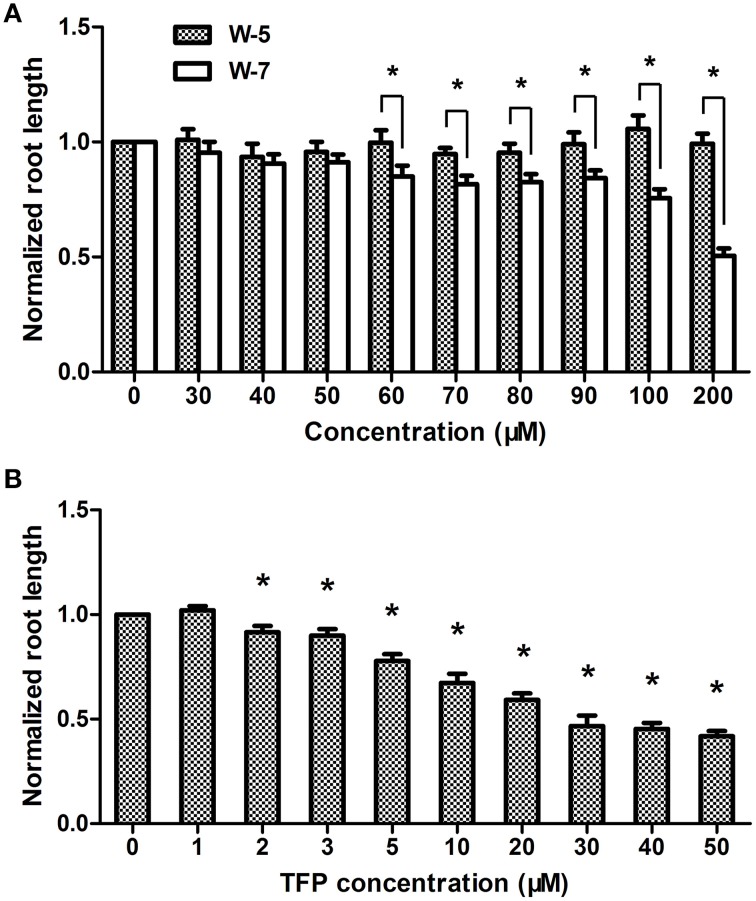
**Effects of CaM antagonist W-7 and TFP at different concentration on primary root elongation**. Wild-type plants were grown on vertically oriented plates containing indicated concentration of W-7, W-5 (no-activity analog of W-7) or TFP and the primary root length was measured after 7 days of cultivation. **(A)** Plot of a dose-response evaluation of W-7 and W-5 (30–200 μM) on primary root length. **(B)** Plot of effects of indicated concentration of TFP (1–50 μM) on primary root length. The results were normalized to the root lengths of untreated samples. Data shown are the average of three independent experiments. Each experiment included at least 30 seedlings. Entries with *p*-values < 0.05 shown with asterisk.

Previously we demonstrated that Ca^2+^ signal is involved in AHL signaling in plant cells (Song et al., 2011). To further analyze the role of CaM in AHL-mediated primary root growth, the effects of different concentrations of CaM antagonists, such as trifluoperazine (TFP) and N-(6-aminohexyl)-5-chloro-1- naphthalene-sulphonamide (W-7), on the length of primary roots was first investigated. It was found that the growth of primary roots in the plants only treated with higher concentration W-7 (Figure 2A) or TFP (Figure 2B) was significantly reduced; however, the length of primary roots did not significantly change in the plants treated with 0–50 μM concentration of W-7 (Figure 2A) or with 0–1 μM concentration of TFP (Figure 2B). W-5 is the dechlorinated analog of W-7 and is often used as control compound for understanding the specificity of W-7. The result shows that no deleterious effect on root elongation was observed for W-5 at the tested concentration (Figure 2A). Therefore, 50 μM for W-7 and 1 μM for TFP have been considered as a threshold concentration and chosen for subsequent experiments.

**Figure 3:**
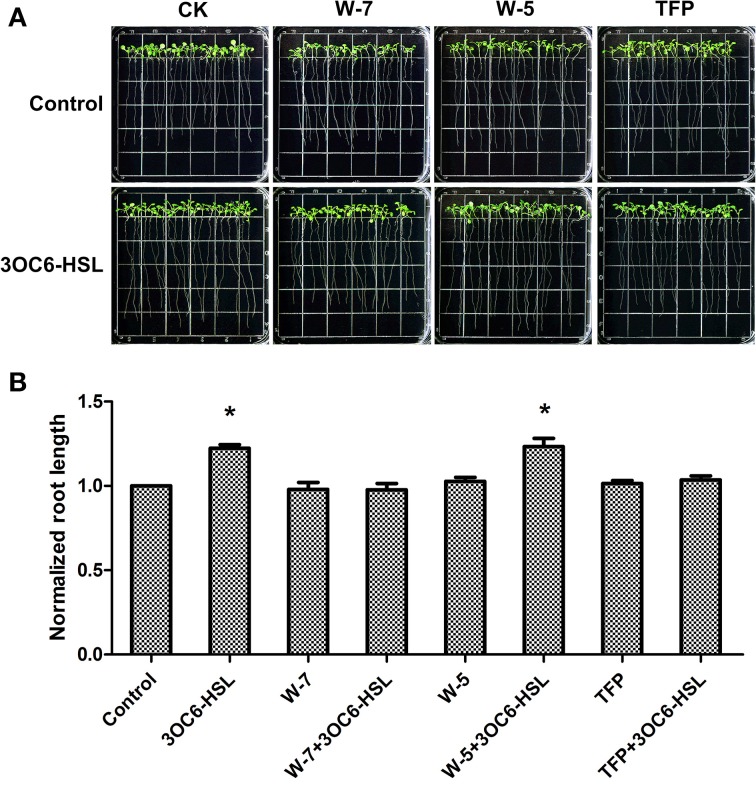
**Impacts of CaM antagonist W-7 and TFP on the growth-stimulating effect of 3OC6-HSL for primary root elongation. (A)** Images of Arabidopsis seedlings grown on vertically oriented plates containing different combination of 3OC6-HSL plus W-7, W-5 or TFP. **(B)** Plot of effects of CaM antagonists on primary root length in presence of 3OC6-HSL. Wild-type plants were exposed to 3OC6-HSL and CaM antagonists W-7, W-5 (W-7 dechlorinated analog, no antagonist activity) and TFP for 7 d and evaluated for variations in primary root length. The plants grown on the plates without addition of 3OC6-HSL, W-7, W-5, and TFP were taken as the control. 1 μM 3OC6-HSL, 50 μM W-7, 50 μM W-5, and 1 μM TFP were applied into media when necessary. The results were normalized to the root lengths of untreated control. Each value is the average of three independent experiments. Each experiment included at least 30 seedlings. Entries with *p*-values < 0.05 shown with asterisk.

Subsequently, we investigated the effect of 3OC6-HSL on root elongation with or without antagonists. Three-day-old seedlings were transferred to 1/2 MS medium plates containing 1 μM 3OC6-HSL and 50 μM W-7 or 1 μM TFP and cultivated for additional 7 days. The plates containing 50 μM W-5 were taken as control for W-7 treatment. The results showed that the 3OC6-HSL-stimulated primary root growth was completely suppressed by addition of 50 μM W-7 while 50 μM W-5 did not affect the effect of 3OC6-HSL on primary root elongation, indicating that the effect of W-7 was attributable to CaM inhibition (Figure 3). Likewise, 1 μM TFP blocked the increase of primary root length in response to 3OC6-HSL as well (Figure 3). These data suggested that CaM has an important role in AHL-stimulated primary root elongation.

### Treatment with 3OC6-HSL promotes the accumulation of the CaM protein

**Figure 4:**
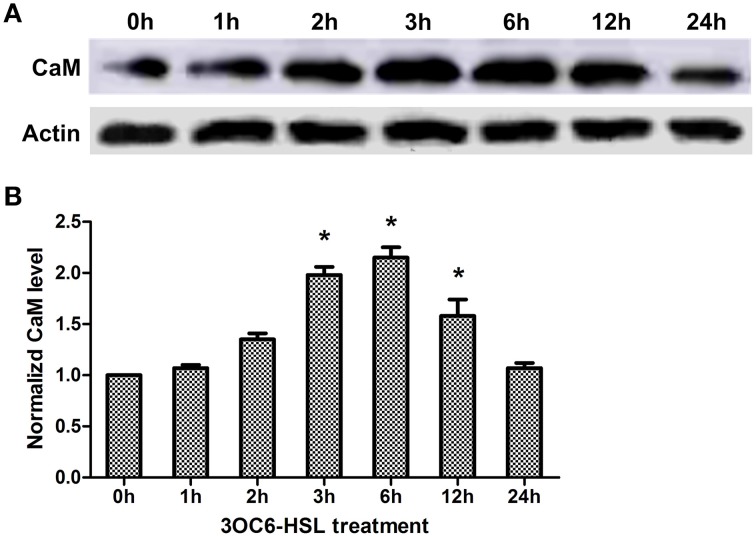
**Effects of 3OC6-HSL on the accumulation of CaM protein in Arabidopsis roots**. The plants were grown in hydroponic Hoagland medium with or without 1 μM 3OC6-HSL. Total protein was extracted from roots at different time interval after 3OC6-HSL treatment. **(A)** The change of CaM protein level detected by western blot. Actin was used as an internal quantification control. **(B)** The change of CaM protein level detected by ELISA using polyclonal antibody against wheat CaM. The results were normalized to the CaM protein level of untreated samples. Each value was the average of three independent experiments. Entries with *p*-values < 0.05 shown with asterisk.

Pharmacological analysis implicates that CaM is required for 3OC6-HSL-mediated primary root elongation (Figure 3). Whether the level of CaM in plant cells is influenced by treatment with 3OC6-HSL has not been resolved. To address this point, we examined the level of CaM protein in Arabidopsis roots reacting to 3OC6-HSL. Total protein was extracted from Arabidopsis roots exposed to 1 μM 3OC6-HSL at different time points and the overall concentration of CaM protein was determined by western blot and ELISA using specific polyclonal antibody against CaM. Western analysis showed that the concentration of CaM protein in roots increased after treatment with 1 μM 3OC6-HSL and reached the maximum at 6 h post treatment (Figure 4A). Similarly, ELISA result indicated that treatment with 1 μM 3OC6-HSL promoted the accumulation of CaM protein and the CaM concentration reached a maximum 2-fold increase after 6 h of treatment with 3OC6-HSL (Figure 4B). These results provide the evidence for a positive regulation of 3OC6-HSL on CaM protein level.

### 3OC6-HSL induces the expression of all nine CaM isoform genes in Arabidopsis

**Figure 5:**
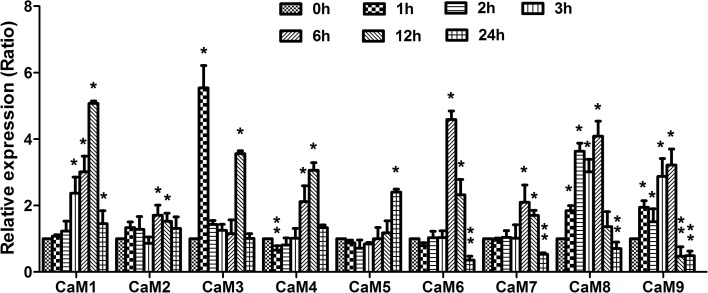
**Effects of 3OC6-HSL on the expression of *CaM* genes in Arabidopsis roots**. The plants were grown in hydroponic Hoagland medium with or without 1 μM 3OC6-HSL. Total RNA was extracted from roots at different time interval after 3OC6-HSL treatment and analyzed by quantitative RT-PCR with specific primers for individual *AtCaM* gene. Values were normalized to the 0 h time point. Data represent the mean from three independent biological replications. Entries with *p*-values < 0.05 shown with asterisk. Relative expression of genes (ratio) >1.5 was considered as up-regulation (single asterisk) and ratio < 0.8 was considered as down-regulation (double asterisk).

The results described above demonstrated that 3OC6-HSL increased the CaM protein accumulation, however the response of *CaM* genes to 3OC6-HSL treatment remains unknown. Therefore, we next investigated the expression profiles of CaM genes after exposure to 3OC6-HSL using qRT-PCR. The Arabidopsis genome contains nine *CaM* genes encoding CaM protein isoforms (Zielinski, 2002). Since a high nucleic acid sequence conservation exists among nine *AtCaM* genes, the primers for each *AtCaM* gene were generally chosen from the 3′ untranslated regions of individual *AtCaM* genes in order to ensure the discrimination between mRNA of genes belonging to highly conserved gene family. Total RNA was isolated from Arabidopsis roots treated with 1 μM 3OC6-HSL and used to perform the real-time RT-PCR. The expressions of all nine *AtCaM* genes were up-regulated significantly by 3OC6-HSL although the extent of induction differed from different *CaM* isoform genes (Figure 5). Compared to the untreated control, a more than 4-fold increase in transcript of *AtCaM1, AtCaM3, AtCaM6*, and *AtCaM8* was observed after treatment with 3OC6-HSL. The mRNA levels of *AtCaM4, AtCaM5, AtCaM7*, and *AtCaM9* genes were increased up to 3-fold by 3OC6-HSL while only 1.7-fold increase was observed in the expression of *AtCaM2* in response to 3OC6-HSL treatment (Figure 5). In summary, these data indicated that 3OC6-HSL induced the expression of all nine *AtCaM* genes with variable degree.

### Mutation of individual *CaM* isoform genes abolish the promotion of primary root length by 3OC6-HSL

**Figure 6:**
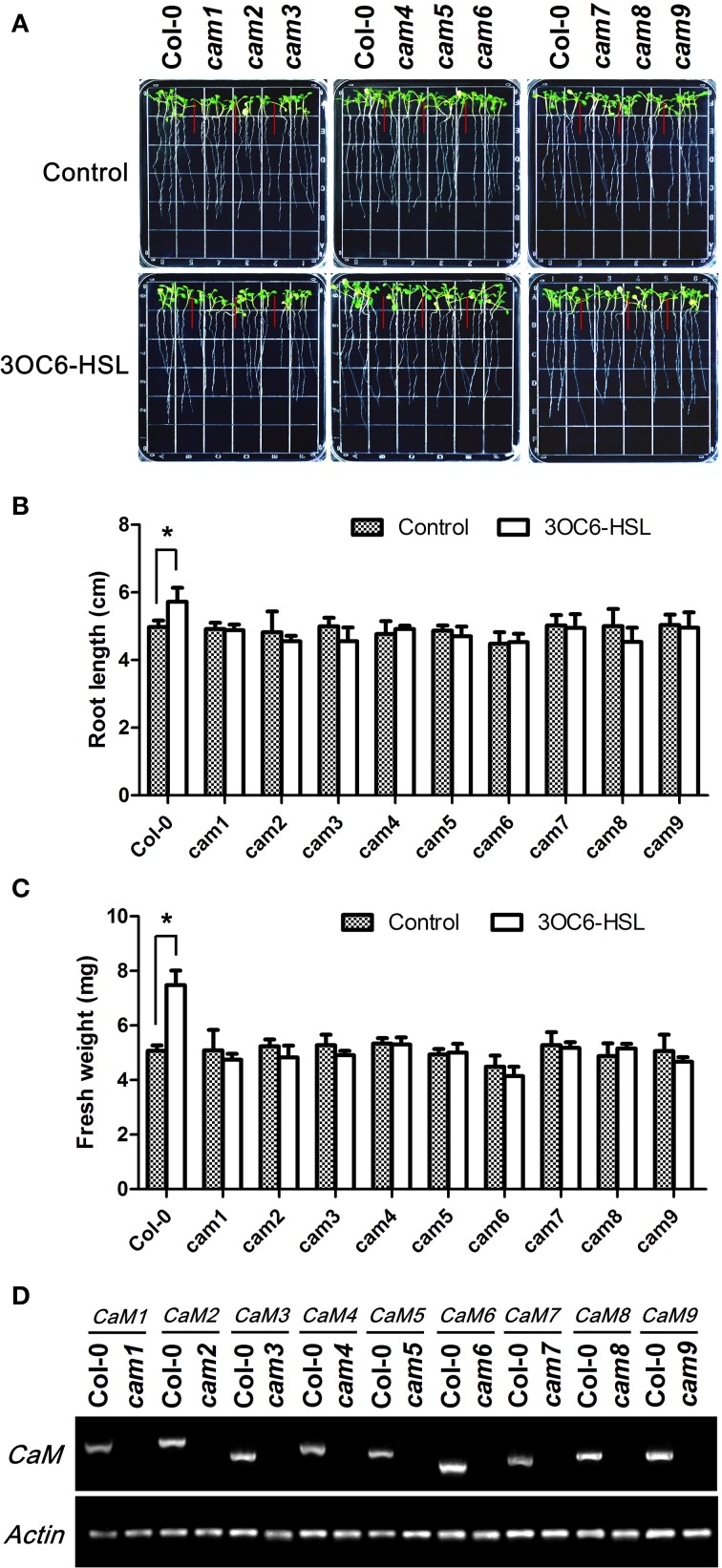
**The stimulating effect of 3OC6-HSL on primary root elongation is diminished in loss-of-function mutants of individual *AtCaM* genes. (A)** Images of Arabidopsis wild-type plants and CaM functional-deficiency mutants *cam1, cam2, cam3, cam4, cam5, cam6, cam7, cam8, and cam9* grown side by side on vertically oriented plates containing with or without 1 μM 3OC6-HSL after 7 days of cultivation. **(B)** Plot of effect of 3OC6-HSL on primary root length in wild-type seedlings and *cam1-cam9* mutant seedlings. **(C)** Plot of effect of 3OC6-HSL on fresh weight in wild-type seedlings and *cam1-cam9* mutant seedlings. Each experiment included at least 30 seedlings for each genotype and treatment. Data shown are the average of four independent experiments. Entries with *p*-values < 0.05 shown with asterisk. **(D)** Identification of CaM null mutants by RT-PCR. Total RNA was extracted from the leaves of one-month-old wild type (Columbia, Col-0) and CaM mutants (cam1-cam9) seedlings and RT-PCR were done to amplify CaM1, CaM2, CaM3, CaM4, CaM5, CaM6, CaM7, CaM8, and CaM9 genes both from wild type RNA and from corresponding CaM mutant RNA. Actin was used as the endogenous reference gene.

The available pharmacological and molecular evidence showed that CaM might be involved in primary root elongation regulated by bacterial AHL, but direct genetic evidence for the participation of CaM is still missing. To solve this question and pursue the specificity of different AtCaM isoform in participation in AHL signaling in plant cells, we compared the primary root elongation in response to 3OC6-HSL between wild-type and the T-DNA insertional mutant lines of individual *AtCaM* gene. All mutants for all nine *AtCaM* genes are confirmed to be homozigous lines by antibiotic resistance screening and RT-PCR (Figure 6D) and exhibit no observed phenotypic differences compared with wild-type plants under normal growth conditions (Figure 6A). As shown in Figure 6, the observed increased root elongation upon 3OC6-HSL exposure in wild-type plant was impaired in all mutation lines of nine *AtCaM* genes. The length of primary roots in *cam1, cam2, cam4, cam5, cam6, cam7*, and *cam9* after treatment with 3OC6-HSL was similar to that of untreated plants of respective mutants (Figures 6A,B). The primary root in *cam3* and *cam8* after contact to 3OC6-HSL exhibited even slightly shorter than that of untreated respective mutants, however no significant difference was found between treatment and untreatment with 3OC6-HSL after statistical analysis (Figure 6B). Additionaly, we measured the effects of 3OC6-HSL on the fresh weight of seedlings of wild-type and the individual *AtCaM* mutants. An obvious increase in the fresh weight of wild type seedlings was observed after treatment with 3OC6-HSL, whereas the fresh weight of seedlings of all nine *AtCaM* mutants was similar between treated and untreated plants (Figure 6C). Although the conclusion for role of AtCaM in AHL signaling at genetic level needs to be reinforced by complementary and over-expression analysis, our data provide preliminary evidence that all nine *AtCaM* genes might be involved in plant response to 3OC6-HSL with respect to primary root elongation.

**Figure 7:**
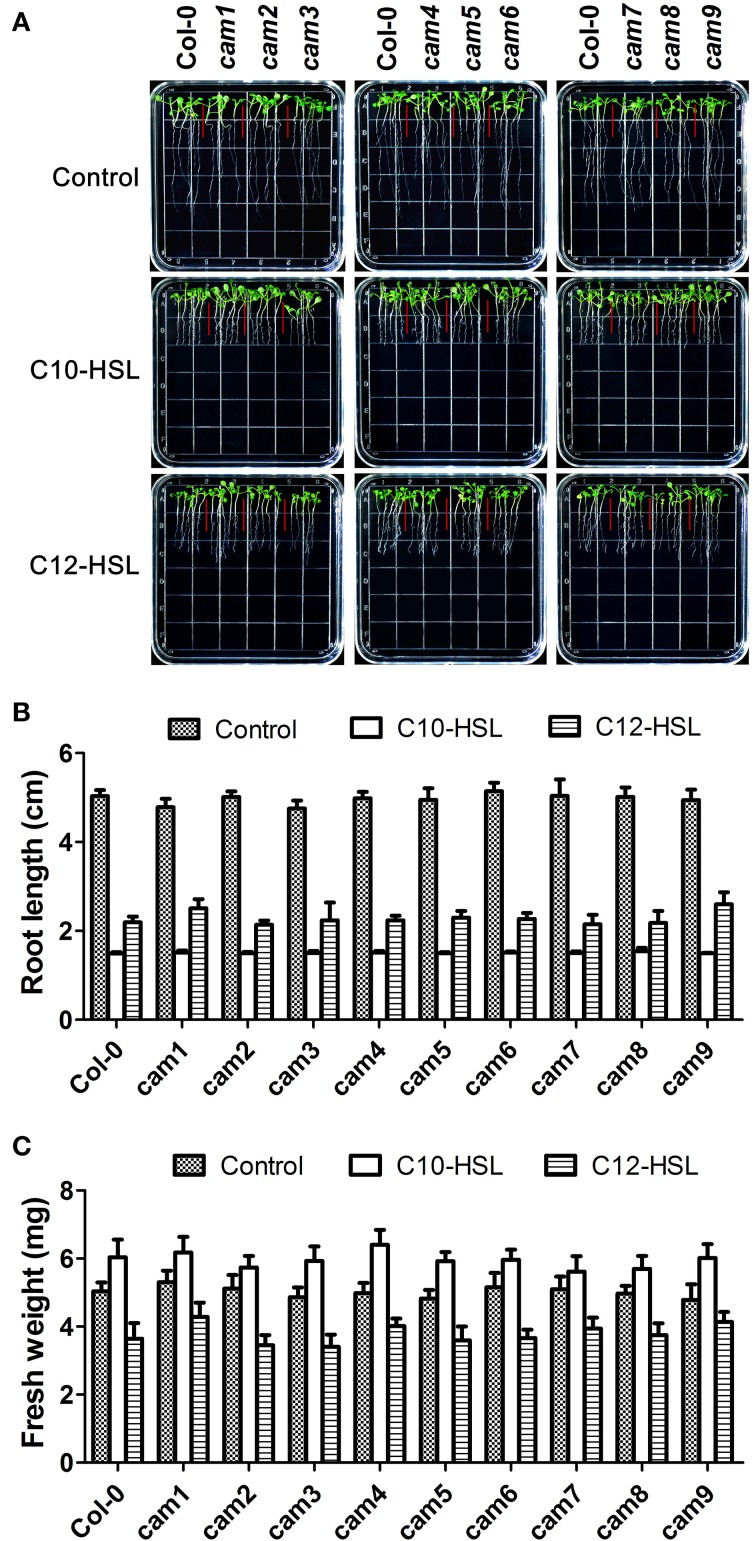
**Effects of C10-HSL and C12-HSL on seedling growth in Arabidopsis wild-type plants and CaM functional-deficiency mutants. (A)** Images of Arabidopsis wild-type plants and CaM functional-deficiency mutants *cam1, cam2, cam3, cam4, cam5, cam6, cam7, cam8, and cam9* grown side by side on vertically oriented plates containing with or without 50 μM C10-HSL or 50 μM C12-HSL after 7 days of cultivation. **(B)** Plot of effect of C10-HSL and C12-HSL on primary root length in wild-type seedlings and *cam1-cam9* mutant seedlings. **(C)** Plot of effect of C10-HSL and C12-HSL on fresh weight in wild-type seedlings and *cam1-cam9* mutant seedlings. Ethanol-treated plants were taken as the solvent control. Each experiment included at least 30 seedlings for each genotype and treatment. Data shown are the average of four independent experiments.

It has been reported that short-side chain AHLs such as C6-HSL and 3OC6-HSL promoted primary root elongation (von Rad et al., 2008; Jin et al., 2012; Liu et al., 2012) while long-side chain AHLs (10–16 carbons) such as C10-HSL and C12-HSL inhibited primary root growth but promoted the formation of lateral root and root hair (Ortiz-Castro et al., 2008, 2011). To investigate if the inhibitory effects of C10-HSL and C12-HSL on primary root elongation, we compared the primary root length between wild-type and the T-DNA insertional mutants of nine *AtCaM* genes after exposure to C10-HSL and C12-HSL. The results indicated that while C10-HSL or C12-HSL inhibited the primary root growth but promoted the lateral root and root hair development of wild type plants at concentration of 50 μM, the responses of the mutants of nine *AtCaM* genes to C10-HSL or C12-HSL were similar as that of wild type plants with respect to the root architecture (Figure 7). These data suggested that AtCaMs might not participated in the regulation of root growth and development by the long-chain AHLs.

## Discussion

As one of the most conserved proteins, CaM is thought to be involved in fundamental cellular processes in both animals and plants. In this study, we provide the pharmacological and molecular genetic evidence for involvement of CaM in plant response to bacterial QS signal, 3OC6-HSL, with respect to primary root elongation. To the best of our knowledge, the connection between CaM and 3OC6-HSL signaling in plant cells has not been reported before.

Plant and bacteria have co-existed for millions of years on the earth and a complex networks consisting of different signaling molecules has been evolved. Accumulating evidence suggest that the mechanisms of plant resistance to pathogens or a plants' responding to beneficial microbes share some commonalities. On the other hand, bacterial pathogens and symbionts depend substantially on QS to colonize and infect their host plants. AHLs are the most common QS signals among Gram-negative bacteria. Like microbial-associated molecular patterns (MAMPs), AHLs might be the indicative for plants that pathogens are in the surroundings to gather themselves for attack or that mutualistes are about to interact with plant roots. Accordingly, Hartmann and coworkers suggested that AHLs are important modulators for interaction between plant and bacteria (Hartmann et al., 2014). However, the early events in the perception and transduction of AHLs in plant cells remain unclear. Recently, we found that several bacterial AHLs can elicit changes in the levels of intracellular Ca^2+^, and 10 μM C4-HSL causes a significant, transient increase in the intracellular concentration of free Ca^2+^ in Arabidopsis root cells (Song et al., 2011). As a mediator protein of Ca^2+^ signal, CaM is activated by binding Ca^2+^, inducing a cascade of regulatory events. However, whether CaM participate the downstream cascade of Ca^2+^ signal triggered by bacterial AHLs remain unknown. Pharmocological assay showed that application of CaM antagonist TFP or W-7 into 3OC6-HSL-treated roots of Arabidopsis abolished the stimulatory effect of 3OC6-HSL on primary root elongation, while W-5, an inactive structural analog of W-7, did not affect the effect of 3OC6-HSL (Figure 3). Control experiments demonstrated that these compounds in the concentration used in this study did not affect the root elongation under normal conditions (Figures 2, 3), indicating that the disappearance of 3OC6-HSL-promoted primary root elongation in presence of TFP or W-7 was due to the inhibited activity of CaM. Then we analyzed the accumulation of CaM protein in plant after treatment with 3OC6-HSL by western blot and ELISA with antibody against CaM protein. It was found that accumulation of the overall CaM protein in Arabidopsis roots increased in response to 3OC6-HSL and reached its maximum 6h after treatment (Figure 4). Thus, the data in this study suggested the involvement of CaM in response of plant to 3OC6-HSL and the role of CaM in 3OC6-HSL-mediated root elongation.

In plant cells, the CaM gene family contains variable members that share a high level of nucleic acid and amino acid sequence identity, and different type of CaM isoforms might has distinct physiological roles. Potato plants have eight CaM genes encoding at least two distinct CaM isoforms, among which the *PCM1* gene was up-regulated upon touch and *PCM6* during tuberization (Takezawa et al., 1995). Among the five divergent soybean CaM genes, *SCaM-4* and *SCaM-5* were transcriptionally activated by phytopathogenic bacterial infection or fungal elicitor application, indicating their involvement in defense against pathogen attack (Lee et al., 1995). Yamakawa et al. (2001) isolated 13 CaM genes from tobacco genome and found that levels of individual CaM genes are differentially regulated both transcriptionally and post-transcriptionally when tobacco are exposed to stresses such as pathogen-induced hypersensitive cell death and wounding. The Arabidopsis genome has nine genetic loci encoding CaM protein isoforms. To determine the role of specific *AtCaM* gene in response of plant to bacterial 3OC6-HSL, the level of transcription for the nine *CaM* genes in root of wild-type seedling grown on medium with and without 3OC6-HSL was measured using specific primers. We found that the expression of all *AtCaM* genes were up-regulated after treatment with 3OC6-HSL, although the level of gene expression was variable between different *AtCaM* members (Figure 5). These data indicated that all these AtCaM isoform genes are 3OC6-HSL -inducible. On the other hand, single *AtCaM* gene knockout did not exhibit any phenotype difference including primary root growth as compared with the wild-type plants under normal growth conditions (data not shown). These observations together with the data of transcription analysis for nine *AtCaM* genes after 3OC6-HSL treatment gave the hints for the existence of functional redundancy between different *AtCaM* members for plant growth.

However, mutation of any single *AtCaM* gene abolished the stimulatory effect of 3OC6-HSL on primary root elongation and the fresh weight (Figure 6). The presence of other *AtCaM* gene in one of *AtCaM* T-DNA insertion mutant seemed not to rescue the increased primary root elongation caused by treatment with 3OC6-HSL. It should be noted that more evidence need to be obtained from the complementary or over-expressing lines of individual *AtCaM* gene. On the other hand, there exist a possibility that some other genes are perhaps required and play additive or complimentary functions. Nevertheless, the molecular and genetic evidence showed the participation of *AtCaM* genes in the response of Arabidopsis to bacterial QS signal 3OC6-HSL. At moment, we could not figure out the specificity of individual *AtCaM* gene in participating the AHL-mediated primary root growth. Liu et al. (2005) found that the expression of nine *AtCaM* genes (*AtCaM 1-9*) was differentially regulated by heat-shock (HS) at 37°C. The expression of *AtCaM3* and *AtCaM7* genes increased, while the expression of *AtCaM2, AtCaM5*, and *AtCaM6* decreased during HS. The levels of *AtCaM1, AtCaM4*, and *AtCaM8* mRNA showed no change during HS. Furthermore, molecular and genetic evidence suggest that endogenous *AtCaM3* is a key component in HS signaling in Arabidopsis. On the other hand, AI-Quraan et al. (2010) reported that *AtCaM1* and *AtCaM5* play important roles in response to HS at 42°C. These inconsistent results implicated the complexity of *AtCaM* participating in the signal transduction of response to the external stimuli.

Previous studies revealed that AHLs exert concentration and acyl-chain length dependent effects on plant primary root growth (Ortiz-Castro et al., 2008, 2011; von Rad et al., 2008; Jin et al., 2012; Liu et al., 2012; Palmer et al., 2014). Generally, AHLs with short-side chain such as C6-HSL and 3OC6-HSL promoted primary root elongation (von Rad et al., 2008; Jin et al., 2012; Liu et al., 2012) while AHLs with long-side chain (10–16 carbons) such as C10-HSL and 3OC12-HSL inhibited primary root length but promoted the formation of laterous root and root hair (Ortiz-Castro et al., 2008, 2011). In addition, even 3OC6-HSL and C6-HSL which exhibit a stimulating effects on primary root growth at concentration below 10 μM showed an inhibitory effects on root length when higher concentration were used (Palmer et al., 2014). In the present study, we found that the prolonged primary root caused by 3OC6-HSL with the tested concentration was the consequence of increased cell division in meristic zone and increased cell elongation in elongation zone (Figure 1). These observation implicated that plants might perceive and respond to different types of AHLs in a different manner. In this study, we provided the evidence that CaM activation might be the early event in 3OC6-HSL signaling in plant cells. However, the genetic evidence shows that CaM may not participates the inhibition of primary root length caused by application of long-chained AHLs such as C10-HSL and C12-HSL (Figure 7). A more thorough understanding of how CaM regulates plant responses to different types of AHL is of great importance. Such investigation is under way.

Our previous work has shown that G-protein-coupled receptor (GPCR) and heterotrimeric G-protein are involved in AHL-mediated primary root elongation of Arabidopsis, and GPCRs might be the candidate receptor for AHL in plant cells (Liu et al., 2012). Moreover, a transient elevation in [Ca^2+^]_i_ concentration could be induced by a number of AHL in plants and the Ca^2+^ influx from the extracellular space into the cells contributed mainly to the increased concentration of cytosolic free Ca^2+^ (Song et al., 2011). Based on our findings herein, we propose a hypothesis that 3OC6-HSL might first be perceived by GPCR and then activate Gα. Gα activation is closely followed by an increase in Ca^2+^ through opening of Ca^2+^ channels in the plasma membrane. The elevated level of cytoplasmic Ca^2+^ then directly activates CaM and promotes the expression and accumulation of CaM. Activated CaM triggers the downstream cascades that ultimately lead to the specific cellular reaction. Further studies are needed to identify the downstream target proteins of activated CaM and to find how CaM activates its target proteins.

## Author contributions

Qian Zhao and Chao Zhang contributed equally to this work and performed the pharmocological analysis and molecular, genetic assay, respectively. Yali Huang, Zhenhua Jia and Haili Li have helped with the acquisition, analysis and interpretation of data for the work. Shuishan Song is responsible for the design of the work.

### Conflict of interest statement

The authors declare that the research was conducted in the absence of any commercial or financial relationships that could be construed as a potential conflict of interest.

## References

[B1] AI-Quraan N. A. Locy R. D. Singh N. K. (2010). Expression of calmodulin gene in wild type and calmodulin mutants of *Arabidopsis thaliana* under heat stress. Plant Physiol. Biochem. 48, 697–702. 10.1016/j.plaphy.2010.04.011 20554213

[B2] Bai X. Todd C. D. Desikan R. Yang Y. Hu X. (2012). *N*-3-oxodecanoyl-L-homoserine-lactone activates auxin-induced adventitious root formation via hydrogen peroxide- and nitric oxide-dependent cyclic GMP signaling in mung bean. Plant Physiol. 158, 725–736. 10.1104/pp.111.185769 22138973 PMC3271762

[B3] Bassler B. L. (1999). How bacteria talk to each other: regulation of gene expression by quorum sensing. Curr. Opin. Microbiol. 2, 582–587. 10.1016/S1369-5274(99)00025-9 10607620

[B4] Braam J. Davis R. W. (1990). Rain-, wind- and touch-induced expression of calmodulin and calmodulin-related genes in Arabidopsis. Cell 60, 357–364. 10.1016/0092-8674(90)90587-5 2302732

[B5] Bradford M. M. (1976). A rapid and sensitive method for the quantitation of microgram quantities of protwin utilizing the principle of protein-dye binding. Anal. Biochem. 72, 248–254. 942051 10.1016/0003-2697(76)90527-3

[B6] Cheval C. Aldon D. Galaud J. P. Ranty B. (2013). Calcium/calmodulin-mediated regulation of plant immunity. Biochim. Biophys. Acta 1833, 1766–1771. 10.1016/j.bbamcr.2013.01.031 23380707

[B7] Chin D. Means A. R. (2000). Calmodulin: a prototypical calcium sensor. Trends Cell Biol. 10, 322–328. 10.1016/S0962-8924(00)01800-6 10884684

[B8] Fuqua C. Parsek M. R. Greenberg E. P. (2001). Regulation of gene expression by cell-to-cell communication: acyl-himoserine lactone quorum sensing. Annu. Rev. Genet. 35, 439–468. 10.1146/annurev.genet.35.102401.090913 11700290

[B9] Fuqua C. Greenberg E. P. (2002). Listening in on bacteria: acyl-homoserine lactone signalling. Nat. Rev. Mol. Cell Biol. 3, 685–695. 10.1038/nrm907 12209128

[B10] Gong M. Li Y. J. Dai X. Tian M. Li Z. G. (1997). Involvement of calcium and calmodulin in the acquisition of heat shock induced thermotolerance in maize seedlings. J. Plant Physiol. 150, 615–621.

[B10a] Götz C. Fekete A. Gebefuegi I. Forczek S. T. Fuksová K. Li X.. (2007). Uptake, degradation and chiral discrimination of N-acyl-D/L-homoserine lactones by barley (Hordeum vulgare) and yam bean (Pachyrhizus erosus) plants. Anal. Bioanal. Chem. 389, 1447–1457. 10.1007/s00216-007-1579-2 17899036

[B11] Hartmann A. Rothballer M. Hense B. A. Schröder P. (2014). Bacterial quorum sensing compounds are important modulators of microbe-plant interactions. Front. Plant Sci. 5:131. 10.3389/fpls.2014.00131 24782873 PMC3986513

[B12] Heo W. D. Lee S. H. Kim M. C. Kim J. C. Chung W. S. Chun H. J.. (1999). Involvement of specific calmodulin isoforms in salicylic acid-independent activation of plant disease resistance responses. Proc. Natl. Acad. Sci. U.S.A. 96, 766–771. 10.1073/pnas.96.2.766 9892708 PMC15211

[B13] Jin G. Liu F. Jia Z. Bian Z. Song S. (2012). Involvement of two G-protein-coupled-receptor candidates, Cand2 and Cand7, in Arabidopsis root growth mediated by *N*-acyl-homoserine lactones, the bacterial quorum-sensing signals. Biochem. Biophys. Res. Commun. 417, 991–995. 10.1016/j.bbrc.2011.12.066 22206669

[B14] Knight M. R. Campbell A. K. Smith S. M. Trewavas A. J. (1991). Transgenic plant aequorin reports the effects of touch and cold-shock and elicitors on cytoplasmic calcium. Nature 352, 524–526. 10.1038/352524a0 1865907

[B15] Lee S. H. Kim J. C. Lee M. S. Heo W. D. Seo H. Y. Yoon H. W.. (1995). Identification of a novel divergent calmoculin isoform form soybean which has differential ability to activate calmodulin-dependent enzymes. J. Biol. Chem. 270, 21806–21812. 10.1074/jbc.270.37.21806 7665602

[B16] Liu F. Bian Z. Jia Z. Zhao Q. Song S. (2012). The GCR1 and GPA1 participate in promotion of Arabidopsis primary root elongation induced by *N*-acyl-homoserine lactones, the bacterial quorum-sensing signals. Mol. Plant Microbe Interact. 25, 677–683. 10.1094/MPMI-10-11-0274 22250582

[B17] Liu H. T. Li B. Shang Z. L. Li X. Z. Mu R. L. Sun D. Y.. (2003). Calmodulin is involved in heat shock signal transduction in wheat. Plant Physiol. 132, 1186–1195. 10.1104/pp.102.018564 12857801 PMC167059

[B18] Liu H. T. Sun D. Y. Zhou R. G. (2005). Ca^2+^ and *AtCaM3* are involved in the expression of heat shock protein gene in Arabidopsis. Plant Cell Environ. 28, 1276–1284. 10.1111/j.1365-3040.2005.01365.x19211698

[B19] Livak K. J. Schmittgen T. D. (2001). Analysis of relative gene expression data using real-time quantitative PCR and the 2(-Delta Delta C(T)) Method. Methods 25, 402–408. 10.1006/meth.2001.1262 11846609

[B20] Mathesius U. Mulders S. Gao M. Teplitski M. Caetano-Anolles G. Rolfe B. G.. (2003). Extensive and specific responses of a eukaryote to bacterial quorum-sensing signals. Proc. Natl. Acad. Sci. U.S.A. 100, 1444–1449. 10.1073/pnas.262672599 12511600 PMC298792

[B21] Miao C. Liu F. Zhao Q. Jia Z. Song S. (2012). A proteomic analysis of *Arabidopsis thaliana* seedling responses to 3-oxo-octanoyl-homoserine lactone, a bacterial quorum-sensing signal. Biochem. Biophys. Res. Commun. 427, 293–298. 10.1016/j.bbrc.2012.09.044 22995300

[B22] Morquecho-Contreras A. Méndez-Bravo A. Pelagio-Flores R. Raya-González J. Ortíz-Castro R. López-Bucio J. (2010). Characterization of *drr1*, an alkamide-resistant mutant of Arabidopsis, reveals an important role for small lipid amides in lateral root development and plant senescence. Plant Physiol. 152, 1659–1673. 10.1104/pp.109.149989 20107026 PMC2832232

[B23] Miller M. B. Bassler B. L. (2001). Quorum sensing in bacteria. Annu. Rev. Microbiol. 55, 165–199. 10.1146/annurev.micro.55.1.165 11544353

[B24] Murashige T. Skoog F. (1962). A revised medium for rapid growth and bio assays with tobacco tissue cultures. Physiol. Plant 15, 473–497 10.111/j.1399-3054.1962.tb08052.x

[B25] Ortega X. Polanco R. Castañeda P. Perez L. M. (2002). Siganl transduction in lemon seedlings in the hypersensitive response against *Alternaria alternata* participation of calmodulin, G-protein and protein kinases. Biol. Res. 35, 373–383. 10.4067/S0716-97602002000300012 12462990

[B26] Ortiz-Castro R. Martínez-Trujillo M. López-Bucio J. (2008). *N*-acyl-L-homoserine lactones: a class of bacterial quorum-sensing signals alter post-embryonic root development in *Arabidopsis thaliana*. Plant Cell Environ. 31, 1497–1509. 10.1111/j.1365-3040.2008.01863.x 18657054

[B27] Ortiz-Castro R. Díaz-Pérez C. Martínez-Trujillo M. del Río R. E. Campos-García J. López-Bucio J. (2011). Transkingdom signaling based on bacterial cyclodipeptides with auxin activity in plants. Proc. Natl. Acad. Sci. U.S.A. 108, 7253–7258. 10.1073/pnas.1006740108 21482761 PMC3084137

[B28] Palmer A. G. Senechal A. C. Mukherjee A. Ané J. M. Blackwell H. E. (2014). Plant responses to bacterial *N*-acyl-L-homoserine lactones are dependent on enzymatic degradation to L-homoserine. ACS Chem. Biol. 9, 1834–1845. 10.1021/cb500191a 24918118 PMC4136694

[B29] Pelagio-Flores R. Ortiz-Castro R. López-Bucio J. (2013). *dhm1*, an Arabidopsis mutant with increased sensitivity to alkamides shows tumorous shoot development and enhanced lateral root formation. Plant Mol. Biol. 81, 609–625. 10.1007/s11103-013-0023-6 23412925

[B30] Quiñones B. Dulla G. Lindow S. E. (2005). Quorum sensing regulates exopolysaccharide production, motily, and virulence in *Pseudomonas syringae*. Mol. Plant Microbe Interact. 18, 682–693. 10.1094/MPMI-18-0682 16042014

[B31] Sander D. Brownlee C. Harper J. F. (1999). Communication with calcium. Plant Cell 11, 691–706. 10.1105/tpc.11.4.691 10213787 PMC144209

[B32] Schenk S. T. Stein E. Kogel K. H. Schikora A. (2012). Arabidopsis growth and defense are modulated by bacterial quorum sensing molecules. Plant Signal. Behav. 7, 178–181. 10.4161/psb.18789 22307043 PMC3405712

[B33] Schenk S. T. Hernández-Reyes C. Samans B. Stein E. Neumann C. Schikora M.. (2014). *N*-acyl-homoserine lactone primes plants for cell wall reinforcement and induces resistance to bacterial pathogens via the salicylic acid/oxylipin pathway. Plant Cell 26, 2708–2723. 10.1105/tpc.114.126763 24963057 PMC4114961

[B34] Schikora A. Schenk S. T. Stein E. Molitor A. Zuccaro A. Kogel K. H. (2011). *N*-acyl-homoserine lactone confers resistance toward biotrophic and hemibiotrophic pathogens via altered activation of AtMPK6. Plant Physiol. 157, 1407–1418. 10.1104/pp.111.180604 21940998 PMC3252169

[B35] Schuhegger R. Ihring A. Gantner S. Bahnweg G. Knappe C. Vogg G.. (2006). Induction of systemic resistance in tomato by *N*-acyl-L-homoserine lactone-producing rhizosphere bacteria. Plant Cell Environ. 29, 909–918. 10.1111/j.1365-3040.2005.01471.x 17087474

[B36] Sieper T. Forczek S. Matucha M. Krämer P. Hartmann A. Schröder P. (2014). *N*-acyl-homoserine lactone uptake and systemic transport in barley rest upon active parts of the plant. New Phytol. 201, 545–555. 10.1111/nph.12519 24102510

[B37] Snedden W. A. Fromm H. (2001). Calmodulin as a versatile calcium signal transducer in plants. New Phytol. 151, 35–66 10.1046/j.1469-8137.2001.00154.x 33873389

[B38] Song S. Jia Z. Xu J. Zhang Z. Bian Z. (2011). *N*-butyrylhomoserine lactone, a bacterial quorum-sensing signaling molecule, induces intracellular calcium elevation in Arabidopsis root cells. Biochem. Biophys. Res. Commun. 414, 355–360. 10.1016/j.bbrc.2011.09.076 21964296

[B39] Taga M. E. Bassler B. L. (2003). Chemical communication among bacteria. Proc. Natl. Acad. Sci. U.S.A. 100, 14549–14554. 10.1073/pnas.1934514100 12949263 PMC304117

[B40] Takezawa D. Liu Z. H. An G. Poovaiah B. W. (1995). Calmodulin gene family in potato: developmental and touch-induced expression of the mRNA encoding a novel isoform. Plant Mol. Biol. 27, 693–703. 10.1007/BF00020223 7727747

[B41] van der Luit A. H. Olivari C. Haley A. Knight M. R. Trewavas A. J. (1999). Distinct calcium signaling pathways regulate calmodulin gene expression in tobacco. Plant Physiol. 121, 705–714. 10.1104/pp.121.3.705 10557218 PMC59432

[B42] von Rad U. Klein I. Dobrev P. I. Kottova J. Zazimalova E. Fekete A.. (2008). Response of *Arabidopsis thaliana* to *N*-hexanoyl-DL-homoserine-lactone, a bacterial quorum sensing molecule produced in the rhizosphere. Planta 229, 73–85. 10.1007/s00425-008-0811-4 18766372

[B43] Yamakawa H. Mitsuhara I. Ito N. Seo S. Kamada H. Ohashi Y. (2001). Transcriptionally and post-transcriptionally regulated response of 13 calmodulin genes to tobacco mosaic virus-induced cell death and wounding in tobacco plant. Eur. J. Biochem. 268, 3916–3929. 10.1046/j.1432-1327.2001.02301.x 11453984

[B44] Yang T. Segal G. Abbo S. Feldmam M. Fromm H. (1996). Characterization of the calmodulin gene family in wheat: structure, chromosomal location, and evolutional aspects. Mol. Gen. Genet. 252, 684–694. 10.1007/BF02173974 8917311

[B45] Zarkani A. A. Stein E. Röhrich C. R. Schikora M. Evguenieva-Hackenberg E. Degenkolb T.. (2013). Homoserine lactones influence the reaction of plants to rhizobia. Int. J. Mol. Sci. 14, 17122–17146. 10.3390/ijms140817122 23965976 PMC3759955

[B46] Zhang W. Zhou R. G. Gao Y. J. Zheng S. Z. Xu P. Zhang S. G.. (2009). Molecular and genetic evidence for the key role of AtCaM3 in heat-shock signal transduction in Arabidopsis. Plant Physiol. 149, 1773–1784. 10.1104/pp.108.133744 19211698 PMC2663753

[B47] Zielinski R. E. (2002). Characterization of three new members of the *Arabidopsis thaliana* calmodulin gene family: conserved and highly diverged members of the gene family functionally complement a yeast calmodulin null. Planta 214, 446–455. 10.1007/s004250100636 11855649

