# Supplementary material for: Involvement of calmodulin in regulation of primary root elongation by N-3-oxo-hexanoyl homoserine lactone in Arabidopsis thaliana
Source: Front Plant Sci. 2015 Jan 13;5:807. doi: 10.3389/fpls.2014.00807 (PMC4292405; doi:10.3389/fpls.2014.00807)
Supplement: Supplementary file 1 [file Table1.DOCX]

**Supplementary Table 1. Primer information of genes investigated in qRT-PCR**

| Gene | Locus | Forward Primer (5’→3’) | Reverse Primer (5’→3’) |
| --- | --- | --- | --- |
| CaM1 | [AT5G37780](http://www.arabidopsis.org/servlets/TairObject?id=132191&type=locus) | GATGATGATGACCTTTTTATGC | TTACACACAAAAGTCACAAACC |
| CaM2 | [AT2G41110](http://www.arabidopsis.org/servlets/TairObject?id=35096&type=locus) | ACAACTTCTTCGGCTTTCTC | TAACCAGCAAAAACCAGCC |
| CaM3 | [AT3G56800](http://www.arabidopsis.org/servlets/TairObject?id=40700&type=locus) | GGACTCGAGGTATGTTTTCTGCTT | TGTTCAGACGCAAAATAGAGCATAA |
| CaM4 | [AT1G66410](http://www.arabidopsis.org/servlets/TairObject?id=29764&type=locus) | TCTACAAAAGAATGATTCGACC | TTATGCCAACGAGAAAGAAAC |
| CaM5 | [AT2G27030](http://www.arabidopsis.org/servlets/TairObject?id=34458&type=locus) | GATGGCAAAGTGAGGAAAC | ATGAAATTTGGGACGAATG |
| CaM6 | [AT5G21274](http://www.arabidopsis.org/servlets/TairObject?id=1000429497&type=locus) | AGCCCACAAAAAGAAACAAG | ATGTCCTAAAGAACGCAACC |
| CaM7 | [AT3G43810](http://www.arabidopsis.org/servlets/TairObject?id=40254&type=locus) | TTTGTTGGTCGTGATTTTTTGG | CGTCACGGACAATAACAAAACC |
| CaM8 | [AT4G14640](http://www.arabidopsis.org/servlets/TairObject?id=128788&type=locus) | CAGGAAAGTGATGCGGAGGA | CACCATCCAAATCTGCCTCCT |
| CaM9 | [AT3G51920](http://www.arabidopsis.org/servlets/TairObject?id=37237&type=locus) | ATCTTTGGCAACGGTGGCATCA | CCATCAAGGTCGGCTTCTCG |
| Actin2 | AT3G18780 | CCAGAAGGATGCATATGTTGGTGA | GAGGAGCCTCGGTAAGAAGA |
