# Supplementary material for: Involvement of calmodulin in regulation of primary root elongation by N-3-oxo-hexanoyl homoserine lactone in Arabidopsis thaliana
Source: Front Plant Sci. 2015 Jan 13;5:807. doi: 10.3389/fpls.2014.00807 (PMC4292405; doi:10.3389/fpls.2014.00807)
Supplement: Supplementary file 2 [file Table2.DOCX]

**Supplementary Table 2. Primer information of genes investigated in RT-PCR**

| Gene | Forward primer (5’→3’) | Reverse primer (5’→3’) | Product (bp) | | |
| --- | --- | --- | --- | --- | --- |
| CaM1 | AAAGAGAGACGACTCTGAATCC | TTTGGTTAAACTCCTAGAAGCA | | 678 |  |
| CaM2 | TCAAGTCCGCATTCGTAGCACA | CCAGCAAAAACCAGCCATGAAT | | 695 |  |
| CaM3 | GATAAATACGGTTGCTCTCTCG | AACAGCATCACTTAGCCATCAT | | 596 |  |
| CaM4 | TTTCACAGCTTCGGAGACTTCA | CTAGGCTCAAATCAAACCCAAG | | 667 |  |
| CaM5 | CCAAAAAATGGCAGATCAGCTC | TCCGTTTAATGGGTGTGAGAGG | | 638 |  |
| CaM6 | ACCGATGACCAGATCTCAGAGT | ATGTCCTAAAGAACGCAACCAG | | 532 |  |
| CaM7 | AGAAGGAACATTCACTCTCACG | CATGCTTAGGATTTCGAGGGAT | | 580 |  |
| CaM8 | AAACCTTGAGCTCCCTCCTCTC | GTGTGAACACGAAGTAGTAACAAC | | 600 |  |
| CaM9 | TAGAGCCAAGAGGTGATGTGTA | CACAAAATGCTAATAAGAGGCAGC | | 599 |  |
| Actin2 | CCAGAAGGATGCATATGTTGGTGA | GAGGAGCCTCGGTAAGAAGA | | 110 |  |
